# Accuracy of Digital Impressions for Veneer Restorations: A Narrative Review and Case Illustration

**DOI:** 10.3390/jcm14113859

**Published:** 2025-05-30

**Authors:** Silvia Rojas-Rueda, Manuel Robles, Margiezel Pagan-Banchs, Pablo Garcia, Hamad Algamaiah, Carlos A. Jurado, Abdulrahman Alshabib

**Affiliations:** 1 Division of Biomaterials, Department of Clinical and Community Sciences, School of Dentistry, The University of Alabama at Birmingham, Birmingham, AL 325233, USA; 2 Department of Prosthodontics, Universidad del Valle de Mexico, Hermosillo 83165, Sonora, Mexico; 3 School of Dental Medicine, Ponce Health Sciences University, Ponce 00716, Puerto Rico; 4 Dental Technology Laboratory, Hermosillo 83165, Sonora, Mexico; 5 Department of Restorative Dentistry, King Saud University, Riyadh 11545, Saudi Arabia; 6 Division of Operative Dentistry, Department of General Dentistry, College of Dentistry, University of Tennessee Health Science Center, Memphis, TN 38163, USA

**Keywords:** intraoral scan, scanners, accuracy, veneers, esthetic zone, ceramics

## Abstract

**Background:** Intraoral scanners have become increasingly popular for final dental prostheses due to their precision, efficiency, and patient-friendly approach. However, their use for capturing final impressions of highly esthetic and thin restorations, such as ceramic veneers, presents unique challenges. Veneer preparations differ significantly from traditional full-coverage crowns, with their smaller and more delicate margins often located at or below the gingival level. This complexity may lead to hesitancy among clinicians regarding the routine use of intraoral scanners in such cases. **Methods:** A comprehensive literature review was performed to evaluate the effectiveness of intraoral scanners for final digital impressions in ceramic veneer restorations within the esthetic zone. Studies published between January 2010 and January 2025 were included in the review. Additionally, a case illustration was provided, detailing the use of an intraoral scanner to capture final impressions for a patient requiring 10 ceramic veneers in the esthetic zone. **Results:** The review demonstrated that intraoral scanning is a reliable technique for veneer impressions, delivering clinically acceptable outcomes. The benefits include enhanced patient comfort, improved workflow efficiency, and a reduction in chairside time. Nonetheless, clinicians must overcome a learning curve with digital technologies and ensure optimal conditions, such as maintaining a dry tooth surface during scanning. The presented case successfully illustrated the use of intraoral scanning, resulting in precise impressions and the fabrication of highly esthetic and functional veneers. **Conclusions:** Intraoral scanning is a predictable and effective method for capturing final impressions for veneer restorations. It offers results comparable to traditional methods while enhancing patient experience and enabling the fabrication of high-quality restorations within an efficient workflow.

## 1. Introduction

Computer-aided design and computer-aided manufacturing (CAD/CAM) have been followed back to the 1970s when computer-aided design and manufacture of dental restorations were first introduced [1]. The original generation systems were designed to eliminate the time-consuming process of crown, inlay, and onlay restorations. During the 1980s, the CAD/CAM dental system was made available to allow clinicians to design and fabricate ceramic restorations chairside, reducing preparation for final restoration time significantly [2,3]. During the 1990s and 2000s, computer technology evolution and 3D scanning propelled CAD/CAM development at an increasing rate, expanding its applications in implant dentistry, removable prostheses, and orthodontic appliances [4]. Intraoral scanners replaced the need for traditional impressions, offering greater comfort to patients and enhanced accuracy [5,6].

CAD/CAM technology offers significant advantages for both patients and clinicians. One of its key benefits is time efficiency, as restorations can be designed, milled, and delivered in a single appointment, eliminating the need for temporary restorations or multiple visits [7]. Additionally, it enhances patient comfort by replacing traditional impression techniques with fast and straightforward digital scanning methods [8]. CAD/CAM streamline the treatment process, improve accuracy, and provide patients with a more efficient and comfortable experience [9]. The precision of computer-aided design contributes to better adaptation, improved esthetics, and increased restoration longevity, resulting in higher patient satisfaction [10]. Furthermore, the CAD/CAM workflow enables clinicians to maintain greater control over each stage of the restoration process, leading to enhanced predictability and improved clinical outcomes [11]. The use of intraoral scanners to scan final impressions for veneers and crowns is growing in popularity because of its benefits such as patient comfort, accuracy, and efficiency [12]. Different studies have indicated that digital crowns provide better or comparable marginal and internal adaptation to the conventional techniques, and digital workflows are found to yield smaller gaps and less adjustment before cementation [13,14]. The intraoral scanner and scan sequence employed could affect the trueness and accuracy of the final restoration, with some scanners having greater accuracy for veneers and single crowns [15]. Additionally, studies confirm that digitally produced crowns, both veneered and monolithic zirconia restorations, are more likely to exhibit greater marginal accuracy than their conventionally impressed counterparts [16]. Overall, digital scanning of crowns and veneers streamlines the restorative procedure, improves adaptation, and assures predictable clinical outcomes [17,18].

Over the past decade, the demand for esthetic dentistry, including veneers, has grown significantly due to advancements in dental materials, adhesives, and digital technologies that enable minimally invasive and highly personalized treatments [19]. Ceramic veneers, in particular, have become increasingly popular because of their superior esthetic qualities, conservative preparation techniques, and excellent long-term clinical performance, with survival rates exceeding 90% over a 10-year period [20,21,22]. The global cosmetic dentistry market continues to expand, and veneers are now widely regarded as a reliable, long-lasting solution for addressing tooth discoloration, minor misalignment, and structural imperfections [23]. Research indicates that both composite and ceramic veneers provide highly satisfactory outcomes for patients seeking to enhance their smiles, with an increasing range of indications as materials and techniques continue to improve [24,25]. In private practices, there is a noticeable rise in patient interest in esthetic treatments, reflecting a broader shift toward patient-centered, minimally invasive dentistry that emphasizes natural and long-lasting results [26].

The use of CAD/CAM technology in veneer production has transformed esthetic dentistry making it possible to achieve accurate, efficient, and minimally invasive treatment procedures. Computer-aided design through digital intraoral scanning makes it possible to achieve very accurate veneer planning and ceramic veneer fabrication, resulting in excellent esthetic outcomes and accurate fit [27,28]. Clinical survival reports are extremely high for CAD/CAM veneer survival of CAD/CAM veneers, and a large retrospective study of lithium disilicate veneers has a cumulative 99.83% survival after several years [29]. Feldspathic and lithium disilicate CAD/CAM veneers have similarly high long-term survival of around 92% after a period of up to 10 years and function as well based on where the veneer sits in the mouth [30,31]. A systematic review confirms that marginal adaptation of CAD/CAM veneers is clinically acceptable and equal to that of conventionally fabricated veneers [32]. In addition, CAD/CAM technology allows for single-visit veneer fabrication and cementation, reducing chair time and improving patient convenience [33].

The aim of this review is to present the current evidence regarding the accuracy of digital impressions for veneer restorations and highlight their clinical relevance through the use of a case study. It tries to provide an overview of the evolution of CAD/CAM technology, the benefits of digital workflows in esthetic dentistry, and the specific requirements in veneer scanning. By way of a review of recent advances, clinical uses, and case study, this review aims to assist clinicians in their knowledge of the predictability, effectiveness, and possible pitfalls of using digital impressions for veneers and ultimately lead to more patient friendly and predictable restorations.

## 2. Materials and Methods

### 2.1. Review Search

Articles published between January 2010 and January 2025 on the topic of final impressions using intraoral scanners for veneer restorations were searched in PubMed, Google Scholar, Scopus, and Web of Science. The following search terms were used in both databases: “digital final impression”, “intra-oral impression”, and “final scan impression”.

The initial PubMed search yielded a total of 251 articles. After reviewing the titles and abstracts, 36 manuscripts were selected. These manuscripts were categorized into three groups: case reports or case studies, in vitro studies, and reviews. The inclusion and exclusion criteria are listed in Table 1. Letters, books, book chapters, case reports lacking details on the dental materials used, and articles without full-text availability were excluded from the review. Only publications discussing the benefits and effectiveness of using intraoral scanners for final impressions for veneer restorations were included in the analysis.

### 2.2. Case Study

A 35-year-old male patient presented to the clinic with the chief complaint of wanting to improve the appearance of his smile. The patient reported having received direct resin composite restorations extending from the maxillary right first premolar to the left first premolar five years ago in another city. However, he expressed dissatisfaction with the current condition of these restorations. A review of the patient’s medical history revealed no significant systemic diseases. The patient reported not taking any medications and denied having any known allergies. Upon clinical examination, the patient was diagnosed with the following:

- Stained and worn resin composite restorations spanning from the maxillary right first premolar to the left first premolar.
- Crack lines visible on the resin composite veneers of the maxillary left central incisor, left lateral incisor, and left canine.

**Figure 1:**
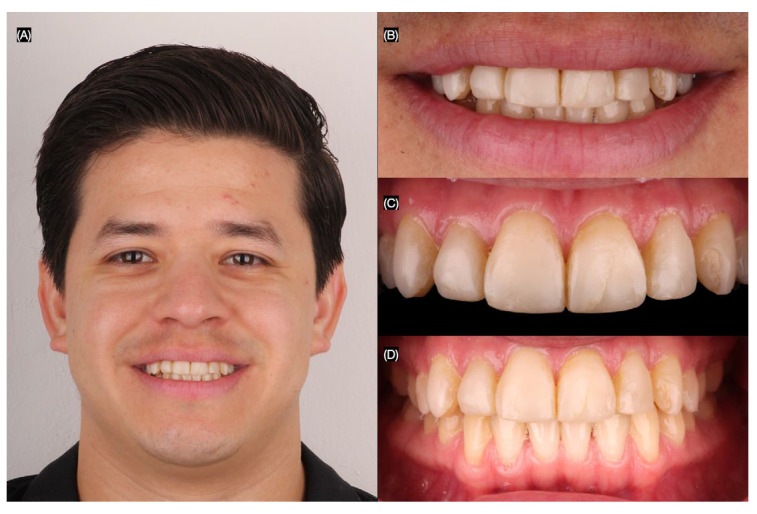
Initial situation. (**A**) Face smiling, (**B**) close-up of the smile, (**C**) intraoral frontal view, and (**D**) dentition in occlusion frontal view.

**Figure 2:**
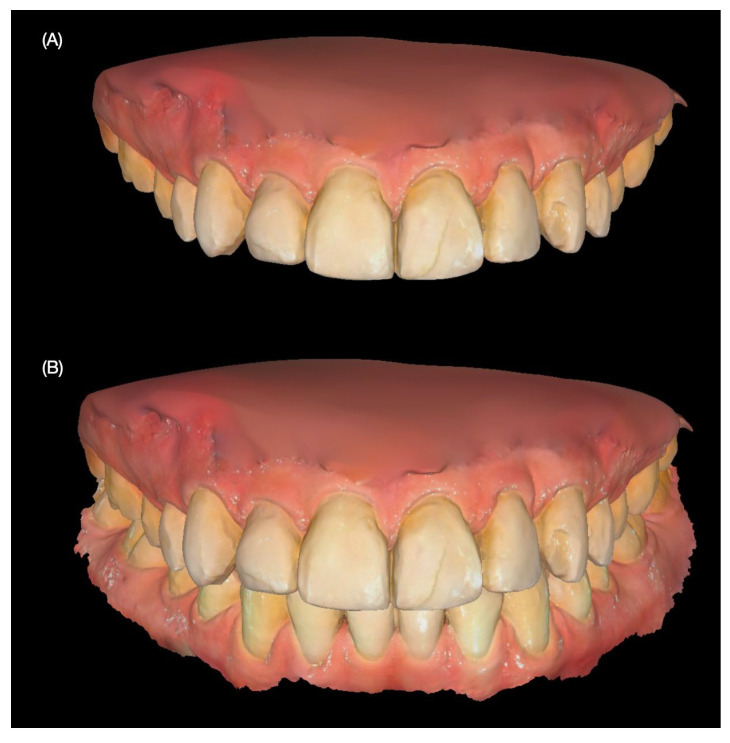
Intraoral initial scan. (**A**) Maxillary frontal view and (**B**) dentition in occlusion.

To document the existing condition and aid in treatment planning, intraoral and extraoral photographs were taken. Additionally, digital intraoral scans of both the maxillary and mandibular arches, as well as scans in maximum intercuspation (occlusion), were obtained (Figure 1 and Figure 2).

This patient was selected for the case illustration due to the need to replace resin composite veneers with ceramic veneers and the plan to obtain the final impression using an intraoral scanner. Although similar cases were reviewed, this patient’s specific clinical requirements, including the extensive number of ceramic veneers needed and their acceptance of an intraoral scan instead of the traditional impression approach, made them an ideal candidate for the procedure.

The patient was offered lithium disilicate veneers extending from the right second premolar to the left second premolar to replace the existing resin composite restorations and cover the entire smile line. The patient agreed to this treatment option. They were informed about the workflow for fabricating the veneers, which included taking the final impression with an intraoral scan and utilizing a combined process involving a printed final model, a printed wax-up of the veneer restorations, and pressable lithium disilicate veneers to achieve highly esthetic results. The patient accepted this approach. Using a diagnostic intraoral scanner (Aoralscan 3, Shining 3D Dental, Hangzhou, China), a digital wax-up (Exocad 2.4, Exocad GmbH, Darmstadt, Germany) was created and a model was printed. Tooth reduction guides and an intraoral putty guide were fabricated with bis-acrylic material (Integrity, Dentsply Sirona, Charlotte, NC, USA). An intraoral mock-up (Integrity, Dentsply Sirona, Charlotte, NC, USA) was provided, allowing the patient to physically evaluate the proposed restoration shape. The patient expressed satisfaction with the result.

**Figure 3:**
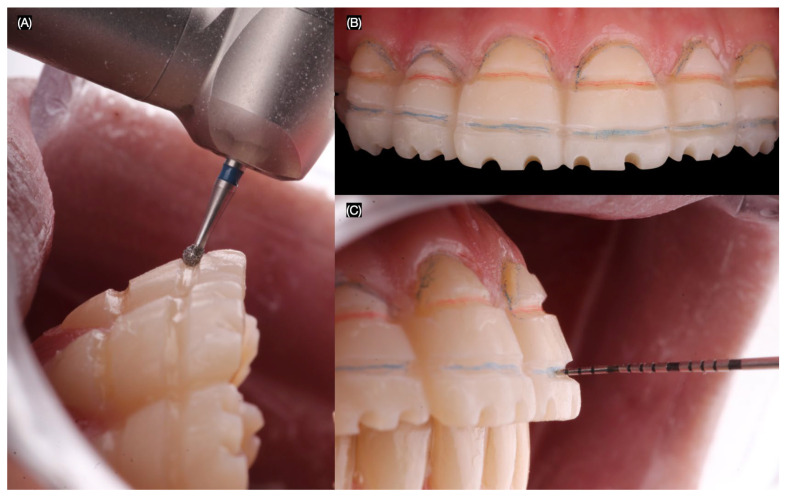
Tooth preparations in mock-up. (**A**) Lateral view creating a tooth reduction groove, (**B**) frontal view of the reduction grooves, and (**C**) measuring the depth of the groove with periodontal probe.

At the subsequent appointment, the same mock-up was placed intraorally, and minimally invasive tooth reduction was performed using a specialized veneer preparation kit (Efficient Veneer Prep Kit, Jota AG, Rüthi, Switzerland). This process began with the creation of horizontal reduction grooves at the gingival and upper third levels, as well as on the incisal edges (Figure 3).

**Figure 4:**
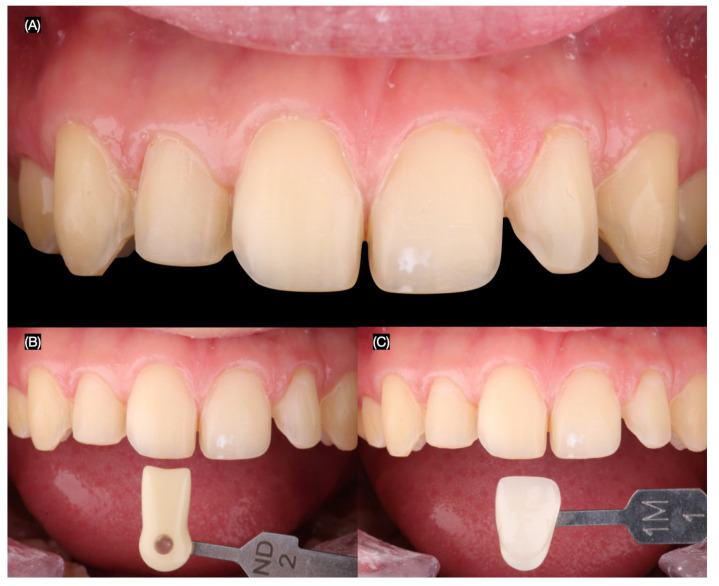
Final preparations and shade evaluation. (**A**) Fina tooth preparations frontal view and tooth shade evaluation with (**B**) dentin shade and (**C**) classic shade guide.

The final tooth preparations were polished using polishing discs (Soft-Lex, 3M, St. Paul, MN, USA) to achieve rounded and smooth surfaces. An intraoral final digital impression (Aoralscan 3, Shining 3D Dental, Hangzhou, China) was taken for both arches, including the dentition in occlusion. Intraoral photographs were captured with dentin shade and classic shade guides to provide detailed information to the dental technician (Figure 4).

**Figure 5:**
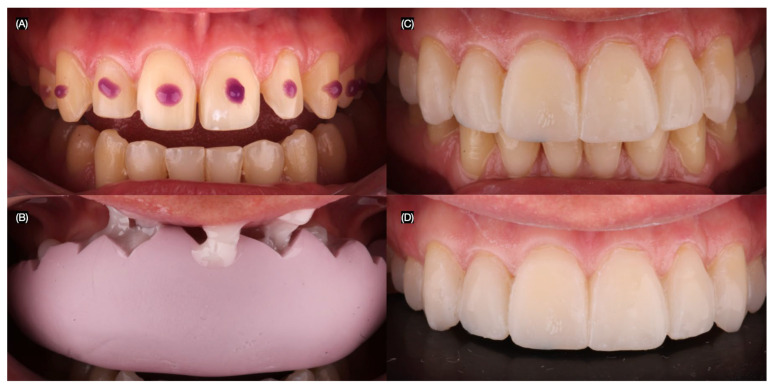
Provisional restorations. (**A**) Spot acid etching on every tooth that received a provisional restoration, (**B**) placement of the interim material with a putty index guide, (**C**) provisional veneer restorations in occlusion, and (**D**) frontal view.

Interim veneer restorations were provided using a spot-etch technique (37.5% phosphoric acid, Gel Etchant, Kerr Dental, Brea, CA, USA) and were splinted and fabricated with bis-acrylic material (Integrity, Dentsply Sirona, Charlotte, NC, USA) in shade A1. The occlusion, as well as protrusive and lateral movements, were evaluated and adjusted as necessary. Finally, the interim restorations were polished (Figure 5).

**Figure 6:**
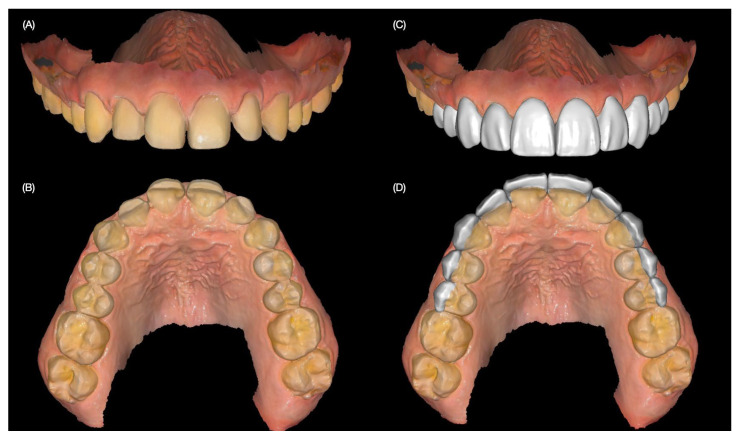
Final scan and design of the restorations. (**A**) Final tooth preparations frontal and (**B**) incisal view, and digital design of the restorations (**C**) frontal and (**D**) incisal view.

Final veneer restorations were digitally designed (Exocad 2.4, Exocad GmbH, Darmstadt, Germany) with the desired contours of the restorations (Figure 6).

**Figure 7:**
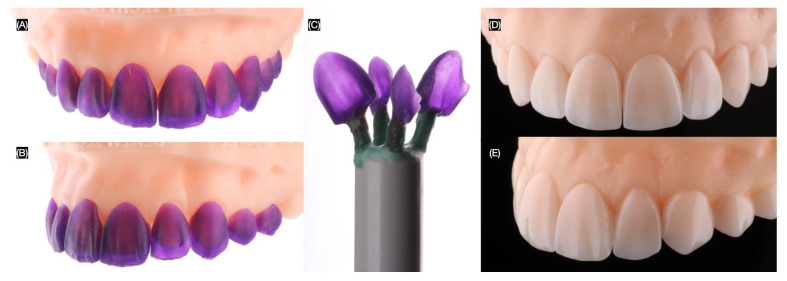
Fabrication of final restorations. Printed wax-up on printed model (**A**) frontal and (**B**) left side view, (**C**) printed wax-up prior pressing, and final veneers (**D**) frontal and (**E**) right side view.

A model of the final intraoral scan was three-dimensionally printed (Anycubic Resin 3D Printer Mono 4K, Anycubic, Shenzhen, China), and the contours of the veneers were printed-out resin (Siraya Tech Cast 3D, San Gabriel, CA, USA) to fabricate them using pressable techniques with lithium disilicate. The margins were evaluated on the printed resin model created from the final impression (Figure 7).

**Figure 8:**
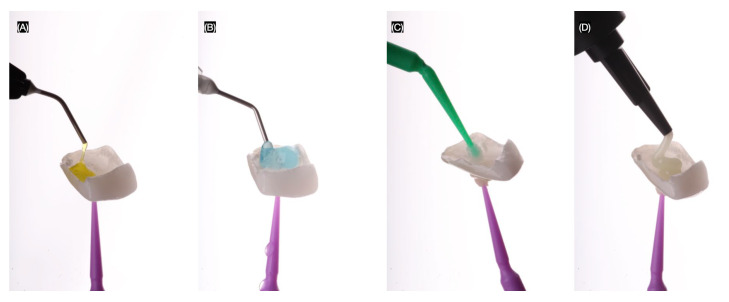
Treatment of the restorations. (**A**) Hydrofluoric acid, (**B**) phosphoric acid, (**C**) silane, and (**D**) resin cement.

The final lithium disilicate restorations (GC LiSi Press, GC, Lucerne, Switzerland) were first treated with hydrofluoric acid (Porcelain Etch, Ultradent, South Jordan, UT, USA) for 20 s, then rinsed and air-dried. The restorations were subsequently cleaned with phosphoric acid (Ultra-etch, Ultradent, South Jordan, UT, USA) for 60 s and then placed in an ultrasonic bath with alcohol and water for 5 min. A silane coating agent (Silane Ultradent, South Jordan, UT, USA) was applied for 60 s. Finally, resin cement (Variolink Esthetic LC, Ivoclar, Schaan, Liechtenstein) was applied before placing the restorations on the teeth (Figure 8).

The teeth were fully isolated prior to the cementation of the veneer restorations. A dental dam (Flexi Dam, Coltene, Altstätten, Switzerland) was placed from the maxillary right first molar to the left first molar using holding clamps, followed by the placement of specialized clamps (B4 Dental Dam Clamps, Coletene, Altstätten, Switzerland) on the teeth receiving the veneer restorations. The sequence began with the two central incisors, followed by the lateral incisors, canines, first premolars, and finally, the second premolars.

**Figure 9:**
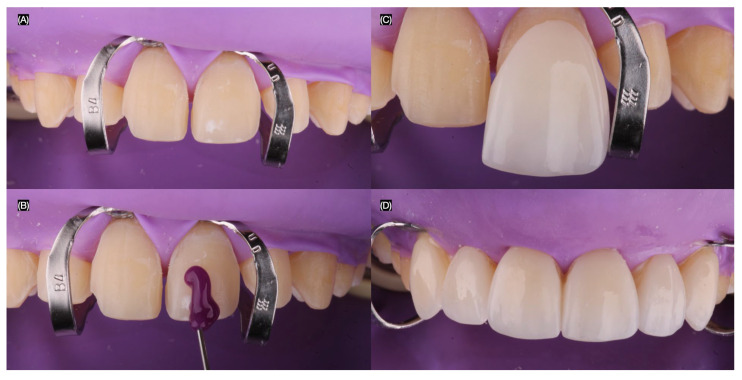
Bonding process. (**A**) Dental dam isolation, (**B**) phosphoric acid application, (**C**) cementation of left central incisor restoration, and (**D**) bonded from canine to canine.

The tooth surface was first cleaned with pumice paste, followed by the application of 29-micron aluminum oxide particles and water (AquaCare Aluminum Oxide Air Abrasion Powder, Velopex, London, England). The surface was then rinsed, air-dried, and etched with phosphoric acid (37.5% phosphoric acid, Gel Etchant, Kerr Dental, Brea, CA, USA) for 20 s, followed by another rinse and air drying. The previously treated lithium disilicate veneer restorations were cemented using resin cement (Variolink Esthetic LC, Ivoclar, Schaan, Liechtenstein). The cemented restorations underwent an initial tack cure (Valo Led, Ultradent, South Jordan, UT, USA) for 5 s, after which the excess cement was removed. A final cure was performed for 20 s on each surface, including the facial, mesial, distal, and incisal aspects (Figure 9).

**Figure 10:**
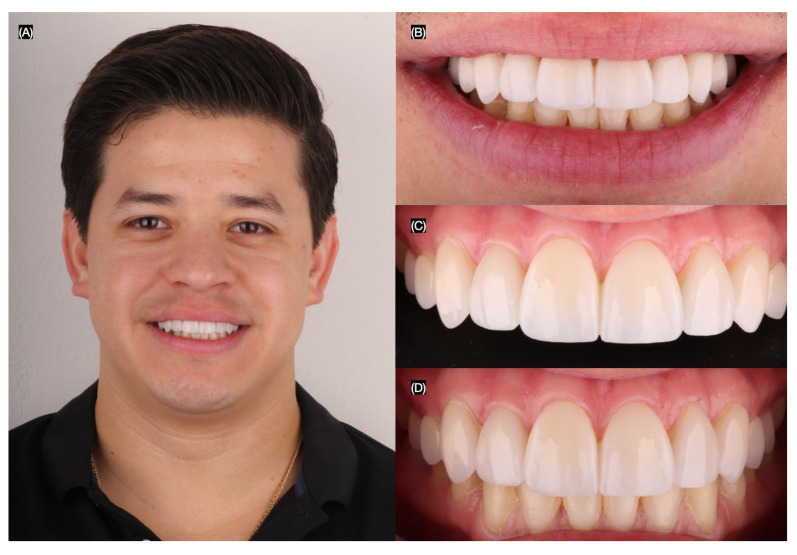
Final bonded restorations. (**A**) Face smiling, (**B**) close-up of the smile, (**C**) intraoral frontal view, and (**D**) frontal view in occlusion.

The patient was fully satisfied with the shade and shape of the final restorations. The occlusion was evaluated and adjusted as necessary. The patient was provided with oral hygiene protocols and instructed to return twice per year for dental prophylaxis and to evaluate the contours, shade, and the surrounding soft tissue of the restorations. Additionally, the patient was provided with an occlusal guard to wear at night to protect the restorations from excessive forces (Figure 10).

At the three-year follow-up appointment, the patient remained satisfied with the lithium disilicate veneer restorations. The restorations retained their original shade and shape, and the periodontal tissues remained healthy. The patient was encouraged to continue practicing good oral hygiene and to consistently wear the occlusal guard.

## 3. Results

### 3.1. Literature Review Outcomes

The literature review conducted provides a wealth of publications addressing the use of intraoral scanners for final impressions in the fabrication of ceramic veneer restorations. Each study was meticulously assessed for methodological quality, ensuring that the findings were robust and clinically relevant. The publications were organized into three distinct tables, categorizing the studies based on their type and focus. The first table includes case studies, case reports, case series, and case illustrations that utilized intraoral scanners for the fabrication of veneer restorations in the esthetic zone. These studies showcased clinical applications, detailing patient outcomes, procedural steps, and the benefits of using digital technology in capturing precise impressions for veneer restorations (Table 2 [34,35,36,37,38,39,40,41,42,43,44,45,46,47]). The second table focuses on laboratory studies evaluating the performance of intraoral scanners. Key metrics such as accuracy, trueness, and precision were assessed, particularly concerning the scanning process for veneer restorations. These studies provided critical insights into the technical capabilities of intraoral scanners and their potential to achieve highly accurate digital impressions (Table 3). Lastly, the third table encompasses reviews, including systematic reviews and meta-analyses, that examined the clinical effectiveness of intraoral scanners for final impressions before the fabrication of ceramic veneer restorations. These comprehensive reviews synthesized evidence from multiple studies, offering a broader perspective on the advantages, limitations, and practical considerations associated with digital impressions. By systematically categorizing the literature, the review highlights the significant role of intraoral scanners in advancing the quality, efficiency, and precision of ceramic veneer restorations in modern dentistry (Table 4).

### 3.2. Case Illustration Outcome

**Figure 11:**
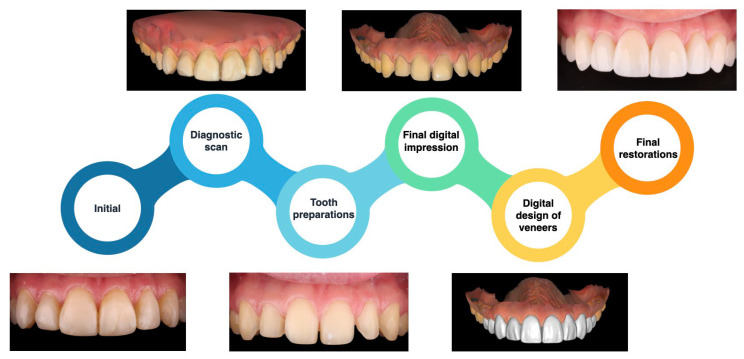
Summary of the clinical steps performed in this case illustration.

The clinical workflow presented in this case illustration outlines the comprehensive steps undertaken to treat a patient with existing resin composite restorations exhibiting stains and fracture lines. This workflow combined novel digital techniques with traditional methods to ensure both precision and efficiency. Following the removal of the old restorations, an intraoral scan was performed to capture a final impression of the prepared teeth. This digital impression served as the basis for generating a three-dimensionally printed model. From this model, a wax-up was milled to reflect the desired contours of the veneers. The wax-up was instrumental in fabricating pressed lithium disilicate veneers with precise dimensions and esthetic attributes. The margins of the final ceramic veneers were thoroughly evaluated on the printed model to confirm accuracy and fit before proceeding with cementation. To ensure optimal bonding and minimize the risk of contamination, the cementation process was carried out under complete isolation using a dental dam. This step is crucial in maximizing the bonding properties and ensuring the longevity of the restorations. The treatment outcome exceeded the patient’s esthetic expectations, successfully addressing the issues of staining and fractures while enhancing the overall smile. A detailed flowchart summarizing the clinical steps involved in this case is provided in Figure 11, offering a visual guide to the workflow. This case illustrates the effectiveness of integrating digital technology with conventional techniques to achieve high-quality, patient-centered restorative outcomes.

## 4. Discussion

### 4.1. Accuracy of Digital Impressions

Whereas traditional impression techniques such as polyvinyl siloxane (PVS) have long been the standard in veneer manufacturing, they tend to be susceptible to error caused by material shrinkage, tray distortion, and dependence on the clinician’s ability [64]. Digital impressions, however, eliminate these parameters through the optical scanning of tooth and structures rather than physical materials [65]. Where conventional methods lack, particularly in deep margins or complex geometries, digital workflows excel, particularly when accompanied adequate gingival management. Intraoral scanners like TRIOS 3 and Primescan have reported trueness of 20–30 μm and accuracy less than 15 μm for single-tooth preparations, above the capability of conventional methods [48]. While analog methods deal with physical distortion, digital technologies have a standard and predictable result with slight variances always being well within the clinically acceptable value of 50 to 120 μm [36]. Ultimately, where conventional impressions reach their limitations, digital workflows begin to deliver their greatest assets, accuracy, efficiency, and reproducibility, in the focus of restorative dentistry [66,67,68,69].

The findings of this narrative review should be interpreted with caution. A recent systematic review and meta-analysis conducted by Revilla-Leon et al. (2025) [60] evaluated the accuracy of intraoral scanner systems for the fabrication of inlay, onlay, and veneer restorations. The review analyzed 34 studies: 17 focused on the accuracy of definitive virtual casts, while the other 17 examined marginal and internal discrepancies. The authors reported that inlay restorations demonstrated better trueness but worse precision in definitive virtual casts compared to onlay restorations. Furthermore, the impression method used did not significantly impact the marginal discrepancies of inlay and onlay restorations. However, the study highlighted the need for further research to evaluate the accuracy of definitive virtual casts for veneer restorations captured by intraoral scanners and to assess the fit of veneer restorations fabricated using intraoral scanning technology. Moreover, another systematic review and meta-analysis evaluated digital versus conventional impression, but for full coverage restorations, the authors assessed ten studies that met the criteria, and the conclusions displayed that low-quality evidence for marginal fit and internal fit suggested similar performance for both techniques, and evidence quality for interproximal contact and occlusal contact was very low and insufficient to draw any conclusions regarding how the impression techniques compared, and the authors concluded that given the uncertainty of the evidence, results should be interpreted with caution [61].

### 4.2. Streamlined File Sharing

Digital workflows enable flawless communication between clinicians and laboratories through standardization of file formats such as STL and PLY [70]. Research validates that digital impressions can be transferred easily to dental laboratories, facilitating rapid case transfer without the need for physical models or shipping, thereby simplifying communication and minimizing errors [71]. Intraoral scanners and CAD/CAM systems accomplish this by generating digital files that are open to a wide range of laboratory software, allowing for interoperability and easy collaboration [72]. The literature highlights that the use of standard digital file formats like STL allows compatibility across between various scanning and manufacturing technologies, yet again bringing flexibility to the workflow and reducing manual adjustments [57,58,59,60,73]. This frictionless digital exchange not only accelerates the speed and accuracy of restorative workflows but also enables better clinical outcomes and patient satisfaction [74].

### 4.3. Expedited Process

The application of CAD/CAM technology in restorative dentistry has significantly accelerated the process of fabrication of restorations such as veneers and crowns compared to traditional procedures [75]. Research has been shown that digital workflows begin with intraoral scanning, which records precise impressions much faster than their analog counterparts [76]. Studies have shown that digital impressions are more time-efficient and better accepted by patients compared to conventional polyvinyl siloxane impressions [63]. The entire digital workflow-scanning, design, and milling process can often offer a final restoration in a single visit. Fewer visits and direct placement benefit patients, while clinicians are able to rapidly change designs and directly communicate with laboratories [59]. Systematic reviews confirm that digital workflows make processes more efficient and maintain or even improve on restoration fit and accuracy [58]. Elimination of physical models and manual procedures reduces errors and remakes, making the process more reliable and patient-friendly. CAD/CAM usually allow delivery of high-quality, esthetic restorations in a more efficient way than ever [77].

### 4.4. Patient Comfort

A number of studies have consistently demonstrated that patients highly prefer digital impressions over traditional polyvinyl siloxane (PVS) impressions. Digital techniques are associated with considerably higher patient satisfaction, primarily due to less discomfort, gag reflex avoidance, and elimination of unpleasant taste or smell commonly associated with traditional materials [66,78]. Clinical trials and network meta-analyses have shown that statistically significantly most patients prefer digital impressions, and even as many as 75% prefer the digital process based on some studies. Digital impressions are also generally thought to be more convenient and quicker, contributing to the patient’s experience even more [79,80]. All of these advantages can be seen using both patient subjective questionnaires and objective scales of acceptance and comfort [81]. Overall, the literature supports that digital impression techniques not only meet clinical requirements but also greatly improve patient comfort and satisfaction levels compared to traditional PVS impressions [82].

### 4.5. Challenges Related to Cost

The expensive initial investment of purchasing an intraoral scanner remains a significant obstacle to the global adoption of digital impression techniques, especially when contrasted with the comparatively low individual cost of traditional PVS impression materials [83]. For example, one study calculated the total cost per digital impression at USD 21.42 per arch compared to USD 29.40 for conventional impressions but noted that the initial purchase cost of the scanner could take over a year to offset in practices with lower patient volumes [84]. Another study found that intraoral scanning presented the lowest overall cost among evaluated workflows but also highlighted that the high initial cost and ongoing expenses for maintenance and software updates could place a financial strain on many dental practices [85]. Even though digital workflows will cost less over the long run with less material and greater efficiency, the startup investment in scanners and associated digital equipment is steep and will not likely be covered immediately, particularly by small practices with limited patient volume [86]. In addition, routine costs of software updates, maintenance, and training also form part of the economic cost, and the transition to computerized systems, thus, is a huge economic undertaking for the majority of clinicians [87].

### 4.6. Technology Learning Curve

One of the biggest initial obstacles to adopting digital technologies like CAD/CAM in dentistry is that there is a steep learning curve in adapting to both hardware and software. Studies have established that dental professionals usually have to spend a few months being trained to acclimatize to the systems, and the period can be described as inefficient and with an increased risk of errors, possibly at the expense of the quality of care during the transition period [88]. For example, learning curve evaluation for 40 subjects showed appreciable differences among software platforms existed with considerable benefits only after having performed the electronic workflow repeatedly 56 times [89]. A cross-sectional study revealed that despite high awareness of CAD/CAM technology (99.5%), many clinicians still cited lack of training and knowledge as key barriers to implementation. Notably, 68.1% of participants advocated for increased CAD/CAM education within undergraduate and postgraduate programs [90]. Among dental students, about 43% initially struggled to work with intraoral scanner software, particularly in difficult areas such as the distal surfaces of posterior teeth. Furthermore, the intricacies and heterogeneity of different systems could lead to inconsistent adoption and underutilization of the full capability of the technology [91].

### 4.7. Limitations of the Review

This narrative review was not performed to a priorly defined and structured protocol of literature search and selection, as systematic reviews require. It was limited to English-language publications in PubMed and industry reports and did not include non-indexed studies. While digital workflows promise much, clinical evidence for veneer survival rates over 5 years is limited. Scanner performance variability and lack of consistent reporting of trueness values contribute to the challenge of comparisons. Future reviews will use systematic and meta-analysis frameworks to estimate accuracy by scanner make and preparation design.

### 4.8. Case Illustration

The presented case illustration serves as a valuable resource for younger clinicians by showcasing a clinical scenario involving a patient with resin composite veneers across all maxillary anterior teeth. The existing veneers exhibited significant staining, as well as crack lines and fractures in certain areas. Dissatisfied with the esthetic outcome of the resin composite veneers, the patient sought a more durable and visually pleasing solution.

The treatment offered involved the replacement of the composite veneers with ceramic veneers. Ceramic veneers were chosen for their superior fracture resistance, immunity to staining, and ability to integrate multiple shades on a single surface, achieving a highly esthetic and natural appearance. After removing the old restorations, tooth preparations were completed, and an intraoral scan of the final preparations was performed. The scan accurately captured the finish lines of the veneer tooth preparations, allowing for the creation of a precise digital file. This file was used to print a three-dimensional model, which guided the milling of a wax-up with the desired contours of the final restorations. The wax-up facilitated the fabrication of pressable lithium disilicate veneers, known for their strength and esthetic properties. The fit of the final veneers was meticulously evaluated on the printed model created from the digital impression, confirming their accuracy. Bonding was carried out under total isolation with a dental dam to prevent contamination and maximize bonding effectiveness. The treatment outcome exceeded the patient’s expectations, addressing their concerns and delivering veneers with exceptional esthetics and durability. This case highlights the advantages of combining advanced digital techniques with traditional workflows to achieve superior restorative results.

### 4.9. Take-Home Message

Although the literature includes clinical case reports demonstrating successful use of intraoral scanners for final impressions in the fabrication of veneer restorations, clinicians should exercise caution. Systematic reviews on this topic highlight the limited availability of data and recommend careful consideration. Several factors can influence the accuracy of intraoral scans, including the calibration of the scanner, the clinician’s experience, and the margin location. Equigingival or supragingival margins are recommended, as subgingival margins may pose significant challenges for scanning. It is important to note that the case illustration presented in this manuscript was performed by dental specialists with extensive experience in the clinical procedure.

## 5. Conclusions

The literature includes several case reports demonstrating the use of intraoral scanners for fabricating veneer restorations in the esthetic zone. However, systematic reviews highlight that data on veneer restorations remain inconclusive due to the limited number of studies available. Clinicians should consider several factors when using intraoral scanners for veneer restorations, such as understanding the learning curve associated with the technology. Additionally, maintaining a dry surface prior to scanning is critical, as scanners cannot accurately capture wet surfaces.

The case illustration presented in this report outlines the steps for a patient scanned intraorally for the fabrication of 10 ceramic veneers. The digital impression facilitated both the design and fabrication of the restorations, significantly expediting the overall process.

## Figures and Tables

**Table 1:** Inclusion and exclusion criteria of the search performed.

Criterion	Inclusion	Exclusion
Time period	Publications available between January 2010 and March 2025	All publications published before January 2010
Language	English	Non-English
Type of articles	All research types, including primary research (e.g., case studies, in vitro studies, and reviews); full text available	Letters, books, book chapters, case reports lacking details on the dental materials used and full text not available

**Table 2:** Summary of clinical case reports using intraoral scanning for veneer restorations [34,35,36,37,38,39,40,41,42,43,44,45,46,47].

Authors/Year	Title	Methods	Results
Zandinejad et al. (2015) [34]	Digital Workflow for Virtually Designing and Milling Ceramic Lithium Disilicate Veneers: A Clinical Report	A 43-year-old man with diastemas and esthetic concerns was treated with multiple lithium disilicate veneers. Digital impressions (Lava COS scanner) were taken after tooth preparation. A fully digital workflow was used for virtual design and remote milling of provisional and final restorations without physical casts. Final veneers were etched, silanated, and bonded with light-cure resin cement.	After one-year follow-up, all veneers exhibited excellent marginal fit, shape, contour, and esthetics. The digital workflow allowed high precision and predictable outcomes, although limitations in artistic detailing with the CAD software were noted.
Lin W.-S. et al. (2015) [35]	Predictable Restorative Workflow for CAD/CAM-Fabricated Ceramic Veneers Utilizing a Virtual Smile Design Principle	A 45-year-old woman received CAD/CAM lithium disilicate veneers on anterior teeth (canine to canine). Treatment included virtual smile design, intraoral scanning (iTero), and digital wax-up.	Intraoral scanning enabled precise digital planning and esthetic predictability. The workflow improved communication with the lab and achieved a successful esthetic result.
Stanley M et al. (2018) [36]	Fully digital workflow, integrating dental scan, smile design and CAD-CAM: case report	47-year-old male; intraoral scanning using Carestream CS 3500 and CS 3600, digital smile design protocol, CAD/CAM fabrication of monolithic lithium disilicate veneers and crowns; minimal invasive preparation.	After treatment, the patient showed stable restorations with no fractures at 6-month follow-up, reported improved comfort and TMJ symptom relief, and adapted well to the increased vertical dimension. The digital workflow was efficient and accurate, but long-term studies are still needed.
Revilla-León M et al. (2019) [37]	Digital tools and 3D printing technologies integrated into the workflow of restorative treatment: A clinical report	58-year-old male; intraoral scanning performed with TRIOS 3 (3Shape). Full digital workflow used: intraoral scans, digital smile design, 3D printed mock-ups, CAD/CAM design of lithium disilicate veneers, milling and DLP additive manufacturing for definitive casts.	The fully digital workflow provided efficient treatment planning, precise esthetics, and predictable outcomes. Intraoral scanning and digital techniques allowed minimal intervention and high patient satisfaction with the final lithium disilicate veneer restorations.
Zhivago P et al. (2022) [38]	A comprehensive technique using digital workflow to improve an unpleasant smile: A clinical report	42-year-old female; intraoral scanning performed using Medit i500 scanner. Smile design created with Smilecloud software, further sculpted with ZBrush software. Three-dimensionally printed mock-up fabricated with SprintRay Pro 55 printer. Lithium disilicate veneers (IPS e.max CAD) were fabricated and cemented.	The fully digital workflow allowed accurate visualization, planning, and realization of the esthetic rehabilitation. The integration of intraoral scanning, facial scans, digital smile design, and 3D-sculpting software provided efficient communication and predictable final esthetic results.
Rathee M et al. (2023) [39]	Anterior esthetic rehabilitation with full and partial veneers using conventional and digital techniques: A case series	Three cases: Conventional impressions were used for veneer cases. In Case 3, a digital workflow was incorporated with a desktop scanner (SMART Open Technologies FARO Europe) and Exocad software to create a virtual diagnostic wax-up and 3D-printed mock-up for zirconia restorations.	The digital workflow allowed accurate design and space evaluation for complex anterior rehabilitation. In all cases, patients were highly satisfied with esthetic results at 1-year follow-up. Digital techniques improved planning precision and restorative outcomes, particularly in cases with complex edentulous spaces.
Figueira J et al. (2023) [40]	Veneer tooth preparation utilizing a novel digital designed workflow: A case report	42-year-old male; intraoral scanning performed to capture digital records for digital wax-up. Virtual guided tooth preparation designed using a BFEP (Bonded Functional Esthetic Prototype). 3D printed preparation guides were used to guide minimal and accurate enamel reduction. Final digital impressions taken after preparation. Veneers fabricated with CAD/CAM (Empress CAD Multi).	The fully digital workflow enabled precise and conservative preparation, preserving maximum enamel. Final restorations achieved excellent esthetics and patient satisfaction. The guided preparation protocol reduced operator error and improved predictability of the outcome.
Pandey et al. (2024) [41]	Trios Lidisi Veneer, Transforming Smiles Using an Intraoral Scanner—A Case Report	A 26-year-old female with anterior spacing was treated with lithium disilicate veneers. Intraoral scanning was performed using a Trios scanner after minimal tooth preparation. Digital models were created and 3D-printed, veneers were milled from lithium disilicate blocks, and restorations were adhesively cemented.	Six-month follow-up showed excellent esthetics, stable restorations, and full patient satisfaction with form, function, and appearance. The digital workflow improved precision, comfort, and efficiency compared to conventional impressions.
Santi M et al. (2024) [42]	Advanced digital planning approach using a 3D-printed mock-up. A case report	45-year-old female; intraoral scanning with Trios 3 (3Shape) after clinical evaluation. Three-dimensional design using Exocad software, digital smile design applied, and a 0.6 mm thick mock-up 3D-printed from resin. Final plan based on the printed mock-up approved by patient.	The fully digital workflow, combining intraoral scanning and 3D printing, allowed an accurate and efficient preview of the esthetic outcome. The mock-up matched patient expectations and guided final restoration planning. The procedure was efficient, predictable, and cost-effective.
Sanchez RLS et al. (2024) [43]	Replacement of unsatisfactory ceramic veneers with the aid of a digital workflow	Case report of a woman in her 30s whose old veneers were replaced using a fully digital workflow. The process included intraoral scanning, digital wax-up, mock-up, tooth preparation, CAD design, and fabrication of leucite-reinforced ceramic veneers.	The digital workflow allowed improved communication, better control of marginal fit, reduced overcontouring, and enhanced esthetic and periodontal outcomes. At 1-year follow-up, the patient showed functional, esthetic restorations with no gingival inflammation.
Osorio-Vélez L.S. et al. (2024) [44]	A Conservative Approach to Ceramic Laminates in the Anterior Region: A Clinical Report	40-year-old female; intraoral scan performed with TRIOS 3 (3Shape) for digital smile design, planning, and fabrication of minimal thickness (0.4 mm) lithium disilicate veneers.	The digital workflow, including intraoral scanning, enabled precise diagnosis, conservative tooth preparation, improved esthetics, and high patient satisfaction.
Al Hamad KQ et al. (2025) [45]	Virtual patient representation with silicone guide and a 3D scanner accessory for a user-friendly facial scanning workflow: A clinical report of smile design and ceramic veneers	23-year-old female; intraoral scanning using Omnicam (Dentsply Sirona) and facial scanning with a Structure Sensor Pro (Occipital Inc) mounted on an iPad. Digital Smile Design (DSD) and 3D facial scans integrated with intraoral scans using a silicone guide for file alignment. Ceramic veneers fabricated using CAD/CAM workflow.	The use of intraoral and facial scans with a simple silicone guide provided an efficient and user friendly digital workflow. Veneers showed excellent fit, esthetics, and patient satisfaction. The protocol proved predictable and cost-effective for full 3D virtual patient generation and esthetic rehabilitation.
Ahmed et al. (2022) [46]	Smile Makeover Utilizing Digital Esthetic Veneers Workflow: A Case Report	A 40-year-old patient seeking to have a new smile to enhance his stained and esthetically unproportional teeth. 3Shape intraoral scanner was used for the diagnosis and final digital impression to fabricate the final veneers from right second premolar to left second premolar.	The use of digital workflow in managing esthetic cases enhanced the treatment predictability and increased the survival and success of the restorations due to the conservation of tooth structure.
do Vale Voigt et al. (2020) [47]	DSDapp use for multidisciplinary esthetic planning	3Shape intraoral scan was used to obtain initial and final impression. Exocad software was used to design the final lithium disilicate veneer restorations	The use of the DSDapp accelerated the initial planning steps and facilitated better communication with the patient and the multidisciplinary team. The use of this application allowed active participation of the patient during the planning process. Additionally, the digital workflow favored greater predictability of the results and achieved the results planned in the DSDapp.

**Table 3:** Summary of laboratory studies evaluating the accuracy of intraoral scanners for tooth-supported restorations including veneers.

Authors/Year	Title	Methods	Results
Boeddinghaus M. et al. (2015) [48]	Accuracy of Single-Tooth Restorations Based on Intraoral Digital and Conventional Impressions in Patients	Clinical study comparing the marginal fit of zirconia copings from three IOSs (3M True Definition, TRIOS, Omnicam) and a conventional impression scanned in lab (3Shape D700) in 49 teeth from 24 patients.	Intraoral scanners (especially 3M and TRIOS) achieved marginal gaps comparable or better than conventional impressions. The study concludes digital IOS can be a reliable alternative when the finish line is visible and dry.
Hategan S.I. et al. (2018) [49]	Powder and Powder-Free Intraoral Scanners: Digital Impression Accuracy	In vitro study comparing the accuracy of two IOSs (Apollo DI—powder and Omnicam—powder-free) used by students, residents, and specialists to scan crown preparations on a typodont.	Powder-free systems (Omnicam) showed higher precision and better marginal adaptation compared to powder systems. Operator experience significantly affected scan quality. Intraoral scanning, especially powder-free, was concluded to offer high clinical accuracy when properly used.
Rotar R.N. et al. (2019) [50]	Trueness and Precision of Two Intraoral Scanners: A Comparative In Vitro Study	In vitro study comparing trueness and precision of Planmeca PlanScan and CEREC Omnicam scanning a prepared molar for an onlay. STL files analyzed with metrology software (Geomagic Control X).	Both scanners showed good accuracy. PlanScan achieved slightly better trueness (48.6 µm) and precision (24.9 µm) than Omnicam (trueness 53 µm, precision 35.6 µm). No statistically significant differences. Intraoral scanning was reliable for onlay preparations.
Diker B., et al. (2020) [51]	Comparing the Accuracy of Six Intraoral Scanners on Prepared Teeth and Effect of Scanning Sequence	In vitro study comparing trueness and precision of six IOSs (Trios 3, iTero, Omnicam, Primescan, Emerald, Virtuo Vivo) for single-crown preparations on canines in a full-arch model. Ten scans per scanner.	Primescan showed highest trueness (25 µm) and precision (10 µm), followed by Trios and Omnicam. Emerald had the lowest accuracy. Scanning sequence significantly affected iTero accuracy. All results were within clinically acceptable range.
Chiu A. et al. (2020) [52]	Accuracy of CAD/CAM Digital Impressions with Different Intraoral Scanner Parameters	In vitro study comparing scan accuracy of 3Shape TRIOS 3 under three resolution settings (Standard, High, Combined) for crown prep finish line on typodont molar. Trueness assessed via Geomagic Control X.	All settings yielded high trueness (<34 µm). No significant difference in accuracy among resolution modes. Accuracy was clinically acceptable; scanner resolution had minimal impact on finish line accuracy.
Son Y.-T. et al. (2022) [53]	Trueness of Intraoral Scanners According to Subgingival Depth of Abutment for Fixed Prosthesis	In vitro study comparing the trueness of two IOSs (i500 and CS3600) at subgingival finish lines (0 mm, 0.25 mm, 0.5 mm, 0.75 mm, and 1 mm) with and without gingival retraction cords.	Without gingival retraction, trueness decreased as subgingival depth increased (>100 μm beyond 0.5 mm). With retraction cord, trueness remained <100 μm at all depths. Gingival retraction improved trueness by ~90%.
Casucci A. et al. (2023) [54]	Accuracy of Four Intraoral Scanners in Subgingival Vertical Preparation: An In Vitro 3D Comparative Analysis	In vitro study evaluating trueness and precision of Trios 3, Medit i700, Vivascan, and Experimental GC IOS in full crowns with subgingival margins (#16, #21).	All IOS showed clinically acceptable accuracy on full abutments (<100 µm RMS). Experimental GC IOS had best precision. Vivascan showed lower performance. Accuracy decreased at subgingival margins.
Giuliodori G. et al. (2023) [55]	Intraoral Scans of Full Dental Arches: An In Vitro Measurement Study of the Accuracy of Different Intraoral Scanners	In vitro study, using 6 IOSs (Medit i700, Primescan, Trios 4, iTero 5D, Omnicam, Dexis IS 3700), scanning an epoxy maxillary model with different strategies. Three hundred scans compared to industrial reference scanner.	Medit i700 and Primescan showed the best trueness and precision (<25 µm). Scanning strategy and operator experience influenced scan time but not clinically significant accuracy. All IOSs achieved acceptable accuracy.
Yang C.-H. et al. (2023) [56]	A Double-Blinded Trial to Compare the Patient Satisfaction and Crown Accuracy of Two Different Intraoral Scanners for the Fabrication of Monolithic Lithium Disilicate Single Crowns	Clinical study (double-blinded crossover trial) comparing Carestream CS3500 vs. MIRDC IOS for scanning posterior tooth-supported crowns in 15 patients (*n* = 40 crowns). Assessed patient satisfaction and crown accuracy (fit, proximal and occlusal contact, general satisfaction).	Both scanners provided high patient satisfaction. Carestream CS3500 showed significantly better accuracy in all crown quality metrics (mean accuracy score 13.3 vs. 6.1, *p* < 0.001). MIRDC IOS was less clinically acceptable.
Park Y. et al. (2023) [57]	Scanning Accuracy of an Intraoral Scanner According to Different Inlay Preparation Designs	In vitro study comparing the accuracy (trueness and precision) of CEREC Primescan for 4 inlay designs on mandibular molars, varying occlusal cavity depth and gingival floor width. Ten scans per group; 3D analysis with GOM Inspect.	The Primescan achieved high accuracy (trueness ~18.5–20.8 μm). Wide and deep cavity designs yielded significantly better trueness. All results were within clinically acceptable range. The study confirms IOS can reliably capture inlay preparations if design allows proper access.
Liu X. et al. (2024) [58]	Accuracy and Efficiency of Digitally Fabricated All-Ceramic Crowns from Conventional Impressions and Intraoral Scans	Single-blind RCT comparing TRIOS intraoral scanning vs. PVS impressions (*n* = 70 crowns). Evaluated marginal/internal fit, adjustment time, occlusal contacts, and dentist satisfaction (VAS).	Intraoral scanning showed significantly better marginal fit (57.9 µm vs. 83 µm), shorter adjustment times, better internal fit, and higher dentist satisfaction (VAS = 8.95 vs. 7.95, *p* < 0.05). Authors concluded IOS was more accurate and efficient than conventional techniques.
Kwon et al. (2024) [59]	Evaluating the Accuracy of CEREC Intraoral Scanners for Inlay Restorations: Impact of Adjacent Tooth Materials	In vitro study evaluating the trueness and precision of Primescan, Omnicam, and Bluecam scanners for inlay restorations on tooth-supported models.	All scanners showed clinically acceptable accuracy. Bluecam achieved the highest trueness. Adjacent gold surfaces improved scanning accuracy compared to zirconia and resin.

**Table 4:** Summary of reviews highlighting the benefits of intraoral scanners for final impression in tooth-supported restorations including veneers in the esthetic zone.

Authors/Year	Title	Methods	Results
Revilla-Leon et al. (2025) [60]	Accuracy of Intraoral Scanner Systems for Fabricating Inlay, Onlay, and Veneer Restorations: A Systematic Review and Meta-Analysis	A literature search was conducted in PubMed, Scopus, Embase, Web of Science, and Cochrane. A total of 34 articles were included in the analysis, with 17 focusing on the accuracy of definitive virtual casts and 17 evaluating marginal and internal discrepancies.	Better trueness but worse precision was observed in the definitive virtual casts of inlay restorations compared to those of onlay restorations. The impression method used did not affect the marginal discrepancy of inlay and onlay restorations. Further studies are needed to evaluate the accuracy of definitive virtual casts for fabricating veneer restorations using intraoral scanners and to assess the fit of the fabricated veneer restorations.
Ahmed et al. (2024) [61]	Mapping the Landscape of the Digital Workflow of Esthetic Veneers from Design to Cementation: A Systematic Review	Twenty articles were chosen to evaluate the digital veneer workflow and the accuracy of digital preparations and cementation guidelines for laminate veneers.	Based on our findings, the digitally fabricated laminate-veneer workflow demonstrated superior predictability and accuracy compared to the conventional workflow.
Pradies et al. (2025) [62]	Comparative Influence of Marginal Design and Digital Scanning Accuracy on the Clinical Longevity of Ceramic Restorations: An Evidence-Based Approach. Consensus Statement From SSRD, SEPES, and PROSEC Conference on Minimally Invasive Restorations	Two systematic reviews with meta-analyses were conducted following PRISMA guidelines. The first review analyzed 15 studies comparing vertical and horizontal finishing lines. The second review included 33 studies on IOS accuracy for inlays, onlays, and veneers.	Vertical and horizontal finish lines demonstrated no significant differences in restoration survival, success rates (65–100%), or periodontal outcomes over 3–7 years. Intraoral scans reliably fabricated single-unit inlay and onlay restorations with high accuracy, but data on veneer restorations remained inconclusive due to limited studies.
Alghauli ET AL (2024) [63]	3D-printed intracoronal restorations, occlusal and laminate veneers: Clinical relevance, properties, and behavior compared to milled restorations; a systematic review and meta-analysis	All studies that assessed 3D-printed partial coverage restorations, including inlays, onlays, laminate, and occlusal veneers, were retrieved. Seventeen records were included in the final review.	Three-dimensionally printed laminate veneers and intracoronal restorations exhibited superior trueness, as well as better marginal and internal fit compared to milled restorations. Some limitations still accompany the resin materials, but this could be overcome by further development of the materials and printing technology.

## Data Availability

Data are contained within the article.
